# A robust motion correction technique for infrared thermography during awake craniotomy

**DOI:** 10.1007/s11548-023-02953-8

**Published:** 2023-05-24

**Authors:** Michael Iorga, Matthew C. Tate, Todd B. Parrish

**Affiliations:** 1 Department of Radiology, Northwestern University, Chicago, IL, USA; 2 Department of Biomedical Engineering, Northwestern University, Evanston, IL, USA; 3 Department of Neurosurgery, Northwestern Medicine, Chicago, IL, USA

**Keywords:** Infrared thermography, Image-guided neurosurgery, Image processing, Motion correction, Awake craniotomy, SSIM

## Abstract

**Purpose:**

Intraoperative infrared thermography is an emerging technique for image-guided neurosurgery, whereby physiological and pathological processes result in temperature changes over space and time. However, motion during data collection leads to downstream artifacts in thermography analyses. We develop a fast, robust technique for motion estimation and correction as a preprocessing step for brain surface thermography recordings.

**Methods:**

A motion correction technique for thermography was developed which approximates the deformation field associated with motion as a grid of two-dimensional bilinear splines (Bispline registration), and a regularization function was designed to constrain motion to biomechanically feasible solutions. The performance of the proposed Bispline registration technique was compared to phase correlation, a band-stop filter, demons registration, and the Horn–Schunck and Lucas–Kanade optical flow techniques.

**Results:**

All methods were analyzed using thermography data from ten patients undergoing awake craniotomy for brain tumor resection, and performance was compared using image quality metrics. The proposed method had the lowest mean-squared error and the highest peak-signal-to-noise ratio of all methods tested and performed slightly worse than phase correlation and Demons registration on the structural similarity index metric (*p* < 0.01, Wilcoxon signed-rank test). Band-stop filtering and the Lucas–Kanade method were not strong attenuators of motion, while the Horn–Schunck method was well-performing initially but weakened over time.

**Conclusion:**

Bispline registration had the most consistently strong performance out of all the techniques tested. It is relatively fast for a nonrigid motion correction technique, capable of processing ten frames per second, and could be a viable option for real-time use. Constraining the deformation cost function through regularization and interpolation appears sufficient for fast, monomodal motion correction of thermal data during awake craniotomy.

## Introduction

Infrared thermography (IRT) is a noncontact technique for measuring the surface temperature of an object [1]. Temperature has a strong relationship with a variety of physiological and pathological processes in the brain [2]. These processes create spatial or temporal temperature gradients on the brain surface which are observable via craniotomy during neurosurgery [3]. For example, brain tumors modify the local vasculature leading to significantly different average temperature than adjacent cortex (spatial gradients) [4, 5]. The patency of a cerebral bypass may be monitored intraoperatively by observing the subsequent warming of the surrounding tissue (temporal gradients) [6, 7]. Intraoperative functional brain mapping may be performed using IRT by measuring the areas where temperature increases when a stimulatory task is performed (spatial and temporal gradients) [8, 9] There is currently potential to construct a neurosurgical diagnostic or monitoring tool based on IRT which addresses one or more of these application areas [10].

Despite these promising early results, an IRT-based device has yet to break into the mainstream neurosurgical arsenal. One of the primary limitations facing IRT is its sensitivity to motion, of which there are several sources. First, the exposed brain exhibits a periodic pulsation which is strong enough to be visible with the naked eye, which originates from the patient’s cardiac and respiratory cycles [11]. Second, intraoperative brain shifting phenomena may slowly change the shape of the brain surface even during craniotomy [12, 13]. Third, patient motion, particularly in awake cases, may move the operating table relative to the camera’s field of view. Motion is problematic because IRT applications often rely on measuring temperature gradients over time and space, rather than absolute temperature [14]. Motion disrupts the spatiotemporal correspondence between pixels which is essential for typical IRT analyses; therefore, motion artifacts can reduce the efficacy of the IRT-based applications mentioned previously [15].

### Related work

Several approaches have been developed for motion correction of infrared thermography data for craniotomy images [15–24]. Selected works are discussed in more detail here.

In Senger et. al., thermal images are preprocessed to enhance the intensity of contours and breathing artifacts, after which a cepstrum-based technique calculates the motion of individual 10 × 10 pixel blocks [18]. The motion across pixel blocks are then combined to estimate motion across the entire image. In Moshaei-Nezhad et. al., the image is again divided into 10 × 10 pixel blocks and motion is corrected in two steps: a phase correlation technique to estimate large displacements, followed by an optical flow technique for subpixel motion [21]. This outperforms the power cepstrum method in both speed and minimizing the root mean-squared error; however, the resulting pixel time series appear flat and it is unclear how much signal information remains intact for downstream analyses.

In Chen et. al., white-light images are collected concurrently with infrared thermal images for the purposes of motion correction [15, 22]. White-light images are coregistered using a combination of feature matching and b-spline approaches. This enables motion correction without affecting the underlying thermal signals. This approach was refined further by Moshaei-Nezhad et. al. by employing Demons registration for faster registration, and this method is likely the current state of the art [23]. Although it is highly accurate, it requires specialized imaging hardware for concurrent collection of white-light and thermal images. Furthermore, this approach has a processing time of about six seconds per image, which is too slow for real-time settings.

### Paper contribution

In this work we present and validate a fast motion correction technique for thermal video data using single-modality intensity-based image registration. The proposed technique models the motion field using a two-dimensional bilinear spline function, defined over a regular grid of control points. The control point positions are adjusted to minimize a cost function which balances similarity to the reference image with biomechanical restraints of brain motion. The paper is organized as follows: first the algorithm design is outlined and optimization approaches are discussed. Lastly, the performance is compared to other algorithms on five minutes of data from ten human patients. Our goal is to create a fast and robust technique that limits the data overfitting effects found in other single-modality images, yielding a practical, near-real-time motion correction solution for the neurosurgical operating room that does not require multimodal imaging.

## Methods

### Proposed method

A motion correction technique for thermal video is proposed based on bilinear interpolation of a two-dimensional spline function (Bispline registration). Consider two thermal images of the same object, one which is a target image and one which is a moved image, which is to be aligned to the target image. Motion can be defined as the vector field which maps each point in the target image to its corresponding point in the moved image. By estimating this field for each frame in a video relative to the initial target frame, a motion correction approach is realized.

Bispline registration estimates the motion vector field using a regular grid of control points. Given a set of motion vectors, one for each control point, the motion vector for each pixel is calculated using bilinear interpolation between the control points. A motion corrected image is generated by applying the inverse of the estimated motion transformation to the moved image. The final motion-corrected pixel values are then computed using bilinear interpolation using the four neighboring pixels. A pixel-wise least squares error metric is calculated by summing the squares of residuals between the target and motion-corrected images. Minimizing the least squares error yields a deformation field which estimates the motion between frames. A complete derivation of the Bispline registration technique is supplied in Appendix A.

Not all vector fields offered as solutions to the least-squares optimization problem described above are physically plausible. The brain is a contiguous and firm physical object, so the order of the pixel positions must be monotonically preserved before and after the transformation is applied. Furthermore, the brain motion being modeled here is relatively smooth over both time and space, so simpler vector field solutions are typically preferred over more complex solutions despite the effect on the least-squares cost function. We address this by adding and weighting a regularization cost function to the objective least-squares cost function. Consider that the regular control point grid is initially composed of many adjacent squares which are perturbed during motion. The sum of squared side lengths of each square is an indicator for the magnitude and nature (stretching, compression) of this perturbation. We therefore define our regularization function as the sum of squared changes in the sum of squared side lengths for each square in the grid (see Appendix A for more detail). The magnitude of this regularization function versus the objective cost function is scaled by a linear constant alpha.

### Motion model optimization

Motion correcting an image therefore constitutes finding a motion vector for each control point which minimizes the overall least-squares error metric. Due to the high framerate of thermal video (30Hz) relative to the expected frequency of the motion (< 1 Hz), we assume that the amount of frame-to-frame motion is relatively small. Although motion correction is a constrained optimization problem to avoid computing deformation fields with overlaps, it is more efficient in practice to treat the problem as unconstrained, and verify that the solutions are feasible. Motion corrected frames with infeasible solutions may be repeated with increased regularization constants until a feasible solution is realized. Objective function minimization is achieved using Newton’s method. The gradient and a sparse Hessian are estimated analytically at each step, specifying a system of linear equations which are solved using the conjugate gradient squared method to specify the subsequent optimization step. The number of Newton steps is treated as a hyperparameter, and the process for selecting it is discussed below.

### Data collection

Ten patients undergoing an awake craniotomy were imaged using infrared thermography. All were recruited from patients undergoing routine surgical resection of gliomas. No changes were made to the routine clinical procedures as a result of participation in the study, including craniotomy size or specific exposure of clinically irrelevant functional areas. All patients were strong candidates for awake functional mapping with direct electrical stimulation, and therefore did not have any significant functional, psychological, or behavioral impairments. All study protocols were reviewed and approved by the institutional review board, and informed consent was obtained for all patients prior to study entry. Patients underwent thermography mapping for at least five minutes, during which a variety of motor or language tasks were performed. During this time the surgical site was not obstructed by surgical instruments, and irrigation of the brain surface by saline solution was withheld. If placement of an absorptive pad was clinically indicated to stop bleeding, the pad was maintained on the brain surface during data collection. If the patients underwent stimulation mapping with direct electrical stimulation prior to thermal data collection, a series of small paper labels indicating positive stimulation sites may also be present on the brain surface during data collection.

**Fig. 1:**
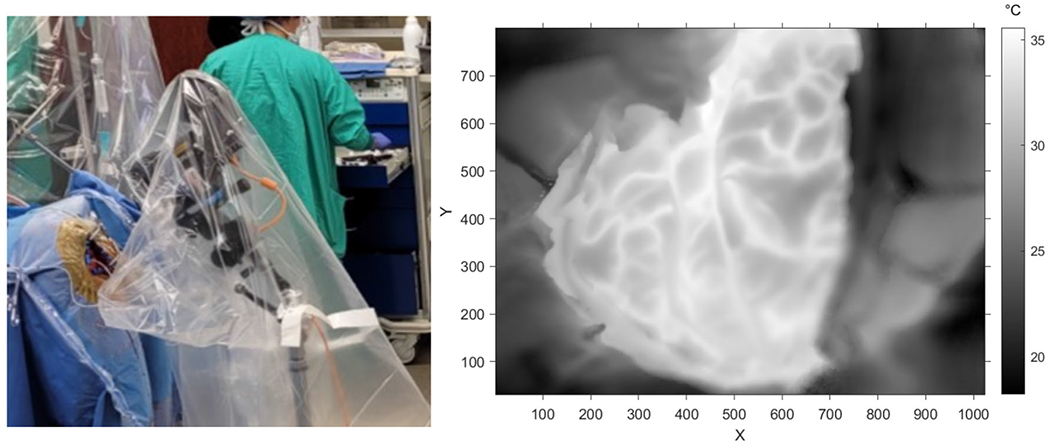
Data collection and craniotomy. Left: The infrared camera setup is shown. The patient’s head is underneath the blue draping, with the lens pointing at the craniotomy site which is surrounded by beige surgical mesh. Right: An example greyscale thermography image of the craniotomy is shown (see temperature colorbar on the right). The craniotomy is the warm, well-focused region in the center in the image. Warm lines correspond to blood vessels within cerebral sulci

Infrared imaging was performed using a FLIR T1020sc thermal camera (resolution 1024 × 768, framerate 30 Hz, NETD < 20 mK). The infrared camera was extended over the craniotomy from a tripod base using a horizontal camera arm (see Fig. 1). The tripod was protected from contact as to not introduce any large camera shake events into the recorded data. For each patient, the camera’s field of view was selected to be in-plane with the craniotomy to maximize the number of pixels measuring craniotomy temperature while respecting a target distance of about 20 cm from the surface. The camera and tripod were wrapped in a sterile surgical polyethylene cover, with a rubber band holding the cover taut over the thermal camera lens.

### Model validation

Validation of motion correction algorithms using operating room data can be challenging as the exact pattern of underlying motion is unknown. We use image quality metrics as an estimate for correct motion estimation. Five minutes of data were collected in each subject, which were downsampled from 30 to 6 Hz for analysis. All data were included in analysis except for the last thirty seconds from patient 9, which exhibited large, fast global motion events and was deemed an outlier.

We compare our algorithm on these ten cases to five other techniques: a phase correlation method, a band-stop filter, Demons algorithm [25], the Horn–Schunck optical flow method, and the Lucas–Kanade optical flow method. Inspired by the study in [26], the applied IIR band-stop filter attenuates frequency bands of 0.5–3 Hz. As these methods are designed for local motion estimation, the phase correlation method was also used as a preprocessing step for these methods. Phase correlation was not applied prior to the proposed method in the initial testing; however, the effect of adding or removing phase correlation is presented below in the analysis of model performance over time. Lastly, it was found during initial testing that the optical flow algorithms performed poorly on longer data intervals, likely due to conflict between thermal data properties and optical flow assumptions. However, the results improved considerably if the optical flow algorithm was applied again to the same data, and in each repeat the results iteratively improved. We treat the number of optical flow repeats as a hyperparameter and discuss its choice alongside all hyperparameters from all the tested methods in Appendix C.

We evaluate the motion correction performance using three metrics of image quality between each frame and the reference image: the mean-squared error (MSE), the peak signal-to-noise ratio (PSNR), and the structural similarity index measure (SSIM) [27]. The formulas for the PSNR and the SSIM are given below:

$$\text{PSNR}=10{\text{log}}_{10}\left(\frac{k^{2}}{\text{MSE}}\right)$$

$$\text{SSIM}\left(x,y\right)=\frac{\left(2\mu_{x}\mu_{y}+C_{1}\right)\left(2\sigma_{xy}+C_{2}\right)}{\left(\mu_{x}^{2}+\mu_{y}^{2}+C_{1}\right)\left(\sigma_{x}^{2}+\sigma_{y}^{2}+C_{2}\right)}$$

For PSNR, k is the largest possible value in the image data type. For SSIM, $\mu_{x}$ and $\mu_{y}$ are the local image means, and $\sigma_{x}^{2},\sigma_{y}^{2},\sigma_{xy}$ are the local variances and covariances for two images x and y. To limit the effect of non-craniotomy pixels on image quality calculation, a craniotomy mask was manually generated for each patient. Each post-registration frame was masked prior to calculating each metric. These craniotomy masks were not used during the motion correction process.

## Results

### Method comparison on clinical data

Several methods were tested on all ten patients: phase correlation, band-stop filtering, Demons registration, Horn–Schunck optical flow, Lucas–Kanade optical flow, and lastly Bispline registration (the proposed method). Hyperparameters were optimized for each method using a grid-based search (see Appendix C for complete description), while trying to keep the processing time for each method around or below one second per frame. Phase correlation used a Gaussian filter with standard deviation of sixteen pixels for signal whitening. The band-stop filter parameters have been set in prior work, so no changes were made. Demons registration used a field smoothing parameter of four pixels, two pyramid levels, and twenty iterations. The smoothing parameter for the Horn–Schunck optical flow method was set to one, and the algorithm was repeated four times. The Lucas–Kanade optical flow noise threshold was set to 0.0625, and the number of repeats was set to three.

The MSE, PSNR, and SSIM were calculated for each frame relative to the initial reference frame. The median results for each patient, technique, and metric are displayed in Tables 1, 2 and 3. All methods were compared to the raw data and to each other on a frame-by-frame basis using a one-sided Wilcoxon signed-rank test for each metric. All methods significantly increased the image quality compared to the raw data across all three metrics (*p* < 0.01). Bispline registration had the highest median MSE and PSNR overall, and across all patients except patient 9, where Demons registration performed best on both metrics. No method had a significantly higher MSE or PSNR as compared to Bispline registration (*p* < 0.01). The Demons registration, Phase correlation, and Band-pass methods significantly outperformed Bispline registration on the SSIM (*p* < 0.01). Although statistically significant differences are present, the absolute difference in overall median IMMSE and PSNR values is relatively small between Demons and Bispline registration, and the overall median SSIM was fairly close for Phase Correlation, band-stop filtering, and demons registration methods. There was no significant difference between the band-pass filter and the phase correlation methods on any metric. Neither optical flow technique performed particularly well overall on any of the metrics.

### Method comparison

**Fig. 2:**
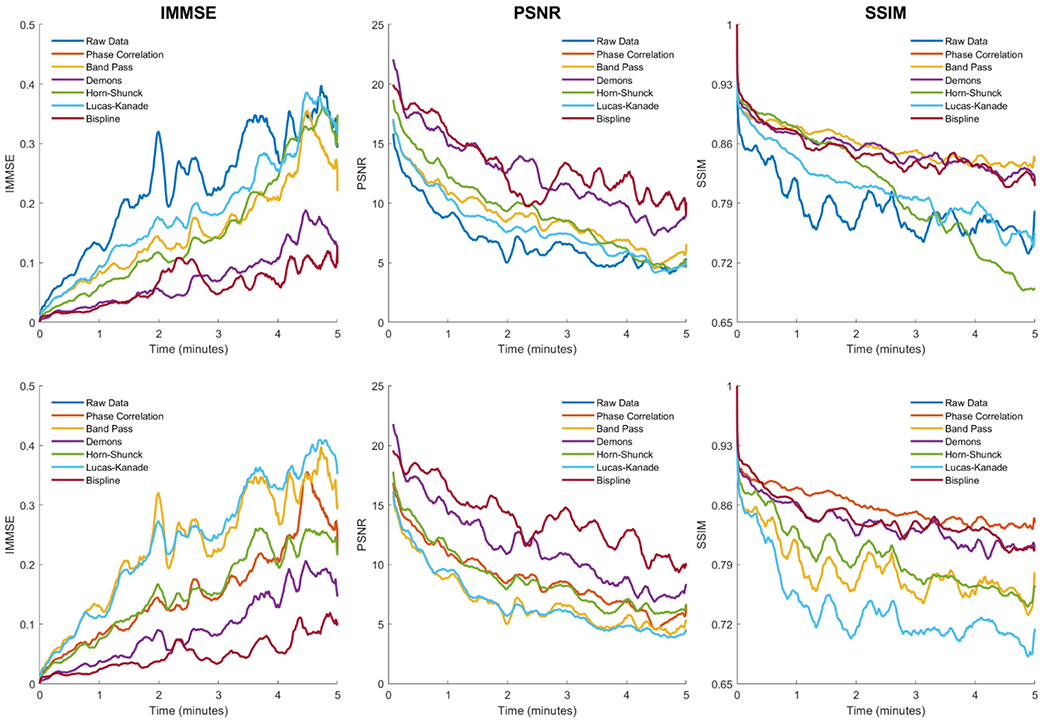
Method performance over time. The medianMSE (left column), PSNR (center column), and SSIM (right column) values are plotted for each method with phase correlation preprocessing (upper row) and without phase correlation preprocessing (bottom row). The phase correlation and raw data are provided as reference in all plots. Due to the minimal effects of band-pass filtering on the image quality estimates, the band-pass line overlaps the phase correlation line in the upper row and the raw data line in the bottom row. The data are smoothed with a 10 s moving average filter for visibility

Motion estimation is more challenging as time goes on due to the cumulative effects of motion and thermal drift. Preprocessing the data with the phase correlation algorithm addresses this by rigidly aligning all frames in the thermal video. We examine the sensitivity of the motion correction methods to time through two analyses. First, the median MSE, PSNR, and SSIM values were calculated across all patients as a function of time. Second, this analysis was repeated but without the phase correlation as a preprocessing step—all methods were tested directly on the raw data. The results of these analyses are shown in Fig. 2.

Overall post-correction image quality decreased over time for all metrics and all methods. This decrease is typically initially sharp over the first thirty seconds and then flattens out. With phase correlation applied, Bispline and Demons registration had the lowest MSE and the highest PSNR values, and the difference versus the other methods is greatly magnified over time. For the SSIM with phase correlation, all methods except for Lucas–Kanade optical flow perform similarly, up until about two minutes at which point the Horn–Schunck method SSIM begins to sharply decrease. Removing phase correlation preprocessed had a large impact on the overall data. All methods except for Bispline registration performed significantly worse on all metrics (*p* < 0.01) when phase correlation preprocessing was removed. When phase correlation preprocessing was added to Bispline registration, it did not improve the MSE or PSNR but did significantly improve the SSIM (*p* < 0.01, median overall SSIM 0.854 vs 0.846).

### Computation time

Balancing the quality of motion corrected images with time it takes to process the data is important in the surgical setting. The processing time per patient per method is shown in Table 4. Average total processing times were 0.825 s per frame for phase correlation, 0.826 s per frame for band-stop filtering. 1.763 s per frame for Demons registration, 1.167 s per frame for Horn–Schunck optical flow, 1.006 s per frame for Lucas–Kanade optical flow, and 0.103 s per frame for Bispline registration. Bispline registration was by far the fastest technique overall, largely but not exclusively due to its lack of dependence on phase correlation preprocessing, which is slower than most methods on their own. When comparing individual method processing time, Bispline registration is approximately at least twice as fast than any other method other than band-stop filtering.

## Discussion

We have proposed and validated a new Bispline registration technique for motion correction of thermal brain images during awake craniotomy. We compared the Bispline registration algorithm to other motion correction algorithms on data from ten awake human patients. As compared to prior work, our comparison is significant for longer data collection intervals, a larger number of frames per patient, and using data from awake patients performing motor or cognitive tasks, all of which may lead to more cumulative motion and thermal drifts over time. While these aspects of our dataset make motion correction more difficult, it represents real challenges in preprocessing neurosurgical thermal data and highlights methods which are relatively robust.

Our analysis suggests that the proposed Bispline registration technique is capable of relatively fast and high-quality motion correction. Each of the other methods tested had weaknesses in one or more areas. Demons registration was competitive with Bispline registration from an image quality standpoint; however, the small increase in SSIM does not justify its use in time-sensitive settings. Phase correlation performed surprisingly well for a global registration technique, producing the best SSIM values across all methods. Phase correlation benefits from highly accurate subpixel global alignment and attenuates some thermal drift through signal whitening. Its strong SSIM performance suggests that correction of local motion in the presence of artifacts can lead to loss of image structure on larger scales. Band-pass filtering the data was not impactful in changing image quality, indicating that there are significant sources of image quality deterioration which lie outside of the 0.5–3 Hz band. These may include patient breathing (~ 0.2 Hz), task-related global motion events (< 0.1 Hz), or turbulent cooling effects of air currents on the brain surface (> 3 Hz).

Although the Horn–Schunck method was not as high-performing at the aggregate patient level, the time analysis in Fig. 2 demonstrates relatively strong performance early on, particularly for the image SSIM. The challenging features of our dataset mentioned previously are destructive for the assumptions of optical flow algorithms, which require small frame-to-frame motion and are intolerant to changes in object brightness. As our patients are awake and engaged in research, the brain surface temperature is inherently unstable due to the impact of functional activation on local blood flow [8]. It is plausible that with correct parameters the Horn–Schunck method could be a viable option for applications with shorter recording times that do not need to be fused together in aggregate. As another optical flow technique, we may form a similar conclusion to performance of the Lucas–Kanade method.

Bispline registration achieves robustness by leveraging known properties of intraoperative motion to constrain the search space for brain motion. The use of control point grids lowers the number of parameters that need to be fit per frame of data. While fewer parameters constrains the complexity of motion that can be estimated, the combination of spatial and grid downsampling effectively creates 16 × 16 image patches on which motion is evaluated. This is opposite of demons registration and optical flow techniques which consider motion on a pixel-by-pixel level. Our approach is similar in this sense to the prior work by Senger et. al. and Moshaei-Nezhad et. al. which analyze motion within 10 × 10 pixel patches and combine the results for global motion estimation [18, 21]. In our work, the synthesis of patch information is performed by the regularization functions, which guide the algorithm toward biomechanically plausible solutions. These regularization functions in conjunction with large pixel patches may decrease sensitivity to textureless regions by extrapolating motion from strongly-textured areas.

### Limitations and future work

While constraining the estimated brain motion leads to improved robustness, it simultaneously requires the user to understand the nature and extent of brain motion in the data. Repeating the parameterization approach as in Appendix B may be necessary before applying the algorithm to new datasets. Additional limitations of our approach may include large amounts of camera shake in which the algorithm may be slow to find the solution and return an under-corrected image. This was seen in Patient 9, which had large amounts of global motion as demonstrated by the relatively strong performance of the phase correlation technique and relatively weak performance of Bispline registration. If this becomes problematic it may be desirable to pre-align the data frames with a phase correlation method before progressing to nonrigid registration, as performed for the other methods and in Moshaei-Nezhad et. al. [21].

A further limitation is the high patient-level variance, as seen in Tables 1, 2 and 3. Patients three, six, and nine had some of the lowest initial image quality, but this only improved in patients three and nine. On inspection, patient six had one of the largest craniotomies in the dataset and the camera was positioned closer to the brain than in other cases. Although this case demonstrated clearly visible motion, the cooling effects of air currents were sharply visible on the cortex and may have limited the performance of motion correction approaches. While other patients had gauze pads in the field of view which experience high frequency cooling and reheating in thermography video, these did not seem to substantially alter algorithm performance. The final thirty seconds from Patient 9 were omitted from analysis due to especially high motion. This patient was unique in that subsequent testing was performed using electrocorticography, and movement of operating room equipment and personnel in anticipation of this experiment were a likely cause for the increased motion.

Surgical applications of intraoperative thermography would benefit from real-time processing. While the presented technique is fast, capable of processing about 10 frames per second, additional speedups are required to reach the native camera sampling rate of 30 Hz. Parallelization techniques and faster computer hardware would both increase the number of frames processed per second. In addition, the number of optimization steps needed for Bispline registration may decrease as the distance between frames decreases. Finally, our technique uses spatially downsampled images, and decreasing or eliminating downsampling would increase registration quality. The balance between speed and quality will ultimately depend on the analysis following motion correction, and studying these downstream impacts is a clear next step for comparison of motion correction algorithms.

## Conclusion

Motion correction of intraoperative thermography data is a vital preprocessing step to attenuate the effects of artifacts which limit data quality. The primary contribution of our work is a fast, registration-based technique based on estimation of motion fields between video frames using a two-dimensional spline function. Motion correction of thermography data is challenging without the use of additional imaging modalities. Thermal noise can make detection of anatomical landmarks challenging, while intensity-based algorithms are liable to degrade physiological signals in favor of matching pixel values. Our work demonstrates that it is possible to overcome these limitations using additional soft constraints on the motion field.

## Supplementary Material

supp

## Figures and Tables

**Table 1:** Median MSEs by patient and technique

Patient	RD	PC	BS	DR	HS	LK	BR
1	0.082	0.064	0.064	0.021	0.079	0.160	**0.019**
2	0.121	0.081	0.081	0.046	0.071	0.085	**0.028**
3	0.245	0.123	0.123	0.038	0.151	0.125	**0.018**
4	0.220	0.215	0.215	0.087	0.156	0.228	**0.062**
5	0.462	0.378	0.378	0.306	0.318	0.473	**0.196**
6	0.346	0.346	0.346	0.282	0.302	0.346	**0.167**
7	0.209	0.174	0.174	0.088	0.151	0.189	**0.035**
8	0.121	0.105	0.105	0.035	0.076	0.170	**0.024**
9	0.509	0.159	0.159	**0.065**	0.135	0.257	0.312
10	0.775	0.071	0.071	0.039	0.062	0.082	**0.033**
**Median**	0.212	0.125	0.125	0.054	0.116	0.166	**0.036**

The MSE between each frame post-motion correction and the initial reference frame was calculated for the raw data (RD, no registration), phase correlation (PC), band-stop filter (BS), demons registration (DR), Horn–Schunck optical flow (HS), Lucas–Kanade optical flow (LK), and Bispline registration (BR, proposed method). The median MSE by technique and by patient, as well as the overall median across all patients, are shown. The minimum value in each row is bolded

**Table 2:** Median PSNRs by patient and technique

Patient	RD	PC	BS	DR	HS	LK	BR
1	10.84	11.95	11.95	16.78	11.04	7.96	**17.12**
2	9.17	10.89	10.89	13.38	11.47	10.71	**15.58**
3	6.10	9.09	9.09	14.19	8.20	9.02	**17.46**
4	6.57	6.68	6.68	10.60	8.07	6.43	**12.08**
5	3.36	4.22	4.22	5.14	4.98	3.26	**7.07**
6	4.60	4.61	4.61	5.49	5.20	4.61	**7.76**
7	6.81	7.60	7.60	10.54	8.21	7.24	**14.59**
8	9.17	9.78	9.78	14.52	11.17	7.70	**16.14**
9	2.93	7.98	7.98	**11.88**	8.68	5.91	5.06
10	1.11	11.47	11.47	14.11	12.07	10.85	**14.84**
**Median**	6.74	9.05	9.05	12.70	9.35	7.80	**14.43**

The PSNR between each frame post-motion correction and the initial reference frame was calculated for the raw data (RD, no registration), phase correlation (PC), band-stop filter (BS), demons registration (DR), Horn–Schunck optical flow (HS), Lucas–Kanade optical flow (LK), and Bispline registration (BR, proposed method). The median PSNRs by technique and by patient, as well as the overall median across all patients, are shown. The maximum value in each row is bolded

**Table 3:** Median SSIMs by patient and technique

Patient	RD	PC	BS	DR	HS	LK	BR
1	0.848	0.883	0.883	**0.895**	0.761	0.793	0.890
2	0.760	**0.852**	**0.852**	0.844	0.833	0.836	0.845
3	0.709	0.837	0.837	0.906	0.692	0.831	**0.915**
4	0.856	**0.880**	**0.880**	0.818	0.848	0.825	0.799
5	0.662	**0.831**	**0.831**	0.754	0.613	0.687	0.657
6	0.885	**0.899**	**0.899**	0.858	0.880	0.897	0.850
7	0.825	0.875	0.875	**0.883**	0.846	0.850	0.873
8	0.810	0.853	0.853	**0.858**	0.815	0.740	0.846
9	0.663	0.848	0.848	**0.859**	0.849	0.787	0.783
10	0.561	**0.832**	**0.832**	0.804	0.716	0.734	0.794
**Median**	0.788	**0.861**	**0.861**	0.858	0.811	0.814	0.846

The SSIM between each frame post-motion correction and the initial reference frame was calculated for the raw data (RD, no registration), phase correlation (PC), band-stop filter (BS), demons registration (DR), Horn–Schunck optical flow (HS), Lucas–Kanade optical flow (LK), and Bispline registration (BR, proposed method). The median SSIMs by technique and by patient, as well as the overall median across all patients, are shown. The maximum value in each row is bolded

**Table 4:** Processing time statistics by patient and technique

Method	PC	BS	DR	HS	LK	BR
Average	0.825	0.002	0.938	0.342	0.181	0.103
MAD (%)	1.50%	5.52%	2.08%	0.76%	2.09%	3.43%
Max (%)	3.36%	13.01%	6.04%	2.51%	4.43%	3.68%
Total	0.825	0.826	1.763	1.167	1.006	0.103

The processing time was measured for the phase correlation (PC), band-stop filter (BS), demons registration (DR), Horn–Schunck optical flow (HS), Lucas–Kanade optical flow (LK), and Bispline registration (BR, proposed method) algorithms. The average time per frame was calculated per patient, and then averaged across patients. The median absolute deviation and maximum times are shown as percentage increase versus the average time. Finally, for methods where the phase correlation technique was necessary, the phase correlation time is added to the individual method’s average time to yield the total processing time
