# Supplementary material for: A robust motion correction technique for infrared thermography during awake craniotomy
Source: Int J Comput Assist Radiol Surg. Author manuscript; Available in PMC 2024 Feb 16. (PMC10632252; doi:10.1007/s11548-023-02953-8)
Supplement: supp [file NIHMS1937019-supplement-supp.pdf]

Supplementary Information

Appendix A: Method Derivation

Definitions

S is the source image MxN
R is the registered image MxN
T is the target image MxN

F is the scalar cost function
G is the gradient vector (1 + M/k)(1 + N/k)x1
H is the hessian matrix (1 + M/k)(1 + N/k)x(1 + M/k)(1 + N/k)

U is the control point matrix for horizontal components (MxN)
V is the control point matrix for vertical components (MxN)

C is the interpolated matrix for horizontal deformation components (MxN)
D is the interpolated matrix for vertical deformation components (MxN)

x is the continuous variable for the horizontal direction in image space
y is the continuous variable for the vertical direction in image space

i is the discrete index for the horizontal direction in image space
j is the discrete index for the horizontal direction in image space

u is the continuous variable for the horizontal direction in grid space
v is the continuous variable for the vertical direction in grid space

k is the grid downsampling rate

Cost Function

Least squares cost function between the registered and target image

$$F(S,T) = \sum_{i=1}^{M}\sum_{j=1}^{N}(T[i,j] - R[i,j])^2$$

Define the interpolation in terms of the [dx, dy] vector for each position

$$R[i,j] = S[x,y]$$

$$x[i,j] = i + C[i,j]$$
$$y[i,j] = j + D[i,j]$$

Set up corners for bilinear interpolation in the image space:

$$x_1[i,j] = floor(x[i,j])$$
$$y_1[i,j] = floor(y[i,j])$$
$$x_2[i,j] = ceil(x[i,j]])$$
$$y_2[i,j] = ceil(y[i,j])$$

Define the bilinear interpolation constants for the image space

$$A_{11}[i,j] = \left(x_2 - x\right)\left(y_2 - y\right)$$
$$A_{12}[i,j] = \left(x_2 - x\right)\left(y - y_1\right)$$
$$A_{21}[i,j] = \left(x - x_1\right)\left(y_2 - y\right)$$
$$A_{22}[i,j] = \left(x - x_1\right)\left(y - y_1\right)$$

Define a matrix of values for each of the corners (bottom-left, bottom-right etc.)

$$S_{11}[i,j] = S\left[x_1, y_1\right]$$
$$S_{12}[i,j] = S\left[x_1, y_2\right]$$
$$S_{21}[i,j] = S\left[x_2, y_1\right]$$
$$S_{22}[i,j] = S\left[x_2, y_2\right]$$

Define bilinear interpolation for the image space using Hadamard Product

$$R[i,j] \ = A_{11}[i,j] \circ S_{11}[i,j] + A_{12}[i,j] \circ S_{12}[i,j] + A_{21}[i,j] \circ S_{21}[i,j] + A_{22}[i,j] \circ S_{22}[i,j]$$

Next we linearly map each deformation vector onto a bilinear control point grid with a downsampling factor of k.

$$u = \frac{1}{k}i - \frac{1}{2k} = \frac{1}{k}\left(i - \frac{1}{2}\right)$$
$$v = \frac{1}{k}j - \frac{1}{2k} = \frac{1}{k}\left(j - \frac{1}{2}\right)$$

Each new points maps to corner positions along the downsampling grid

$$u_1 = floor(u)$$
$$v_1 = floor(v)$$
$$u_2 = ceil(u)$$
$$v_2 = ceil(v)$$

Control point grid is composed of matrices U and V, which define the interpolation grids for dx and dy, respectively.

$$U_{11}(u,v) \ = U\left[u_1, v_1\right]$$
$$U_{12}(u,v) \ = U\left[u_1, v_2\right]$$
$$U_{21}(u,v) \ = U\left[u_2, v_1\right]$$
$$U_{22}(u,v) \ = U\left[u_2, v_2\right]$$

$$V_{11}(u,v) \ = V\left[u_1, v_1\right]$$
$$V_{12}(u,v) \ = V\left[u_1, v_2\right]$$
$$V_{21}(u,v) \ = V\left[u_2, v_1\right]$$
$$V_{22}(u,v) \ = V\left[u_2, v_2\right]$$

Define the bilinear interpolation constants for the control grid space. These are defined by the image size and downsampling rates, and are always constant.

$$B_{11}[i,j] = \left(u - u_2\right)\left(v - v_2\right)$$
$$B_{12}[i,j] =- \left(u - u_2\right)\left(v - v_1\right)$$

$$B_{21}[i,j] = -\left(u - u_1\right)\left(v - v_2\right)$$
$$B_{22}[i,j] = \left(u - u_1\right)\left(v - v_1\right)$$

Define bilinear interpolation for the control grid space using Hadamard Product

$$C[i,j] \; = B_{11}[i,j] \circ U_{11}[i,j] + B_{12}[i,j] \circ U_{12}[i,j] + B_{21}[i,j] \circ U_{21}[i,j] + B_{22}[i,j] \circ U_{22}[i,j]$$
$$D[i,j] \; = B_{11}[i,j] \circ V_{11}[i,j] + B_{12}[i,j] \circ V_{12}[i,j] + B_{21}[i,j] \circ V_{21}[i,j] + B_{22}[i,j] \circ V_{22}[i,j]$$

There are two practical considerations to mention here.

First integers are poorly handled using the above formulation. This is due to the simplicity of the floor and ceiling operations in the above steps, which approximate the corners for bilinear interpolation but do not strictly handle them. While a more complex function for corner identification would address this, the problem of undefined gradients and hessians would remain. We therefore do a check each time the gradient is calculated to ensure that there are no integers, and define a small value epsilon on which to move each integer value forward prior to further computation.

Second, it is possible for points in the grid to exceed the bounds of the image, where the true values are undefined. There are some options for addressing this. First, if it is known that there is no rigid movement, the algorithm can be defined to maintain the frame. This is probably the easiest solution, as it forces the perimeter values of the control point grid to zero and allows optimization of only center values. However, this behavior is not currently guaranteed. A simple alternative is to use constant extrapolation, where all values outside the grid are extrapolated to the nearest pixel. This is less elegant as it relies on the regularization functions to keep the image together but in practice may perform the best, so it is suggested here.

Gradient

The cost function changes when the registered image values change, which only happens as a result of changes in the control point grid. The control point values are therefore the variables of interest, with which to take the gradient for. There are eight variables for each pixel, corresponding to the eight values defining two-dimensional transformation of each pixel's respective bilinear interpolation square. We begin with an example case for U_{11} (x-value for the bottom left corner) and go from there.

The cost function is a least squares function and has a straightforward derivative.

$$\frac{d}{dU_{11}}F(S,T) = \frac{d}{dU_{11}} \sum_{i=1}^{M}\sum_{j=1}^{N} (T[i,j] - R[i,j])^2$$

$$\frac{d}{dU_{11}}F(S,T) = 2\sum_{i=1}^{M}\sum_{j=1}^{N} (T[i,j] - R[i,j])\frac{dR}{dU_{11}}$$

The interpolated image is a sum of four Hadamard products. The source image values are independent of the deformation and can be treated as constant.

$$\frac{dR}{dU_{11}} = S_{11} \circ \frac{dA_{11}}{dU_{11}} + S_{12} \circ \frac{dA_{12}}{dU_{11}} + S_{21} \circ \frac{dA_{21}}{dU_{11}} + S_{22} \circ \frac{dA_{22}}{dU_{11}}$$

The A matrices are area coefficients for bilinear interpolation. They are the product of an x and y direction distance. Therefore, one will always be constant.

$$\frac{dA_{11}}{dU_{11}} =- \left(y_2 - y\right)\frac{dx}{dU_{11}}$$

$$\frac{dA_{12}}{dU_{11}} =- \left(y - y_1\right)\frac{dx}{dU_{11}}$$

$$\frac{dA_{21}}{dU_{11}} = \left(y_2 - y\right)\frac{dx}{dU_{11}}$$

$$\frac{dA_{22}}{dU_{11}} = \left(y - y_1\right)\frac{dx}{dU_{11}}$$

Note that there are inverses here allowing for a possible simplification in the previous equations by adding together S matrices before computing the product. This decreases the number of Hadamard products that must be performed.

$$\frac{dR}{dU_{11}} = \left(\left(S_{21} - S_{11}\right) \circ \left(y_2 - y\right) + \left(S_{22} - S_{12}\right) \circ \left(y - y_1\right)\right) \circ \frac{dx}{dU_{11}}$$

Furthermore, x is defined by C, in which U_{11} appears as the Hadamard product with B_{11}.

$$\frac{dx}{dU_{11}} = \frac{dC}{dU_{11}} = B_{11}[i,j]$$

We now substitute and attempt to simplify by merging like terms, preferring matrix additions to Hadamard products.

$$\frac{dR}{dU_{11}} = \left(\left(S_{21} - S_{11}\right) \circ \left(y_2 - y\right) + \left(S_{22} - S_{12}\right) \circ \left(y - y_1\right)\right) \circ B_{11}$$

$$\frac{dF}{dU_{11}} = 2(T - R) \circ \left(\left(S_{21} - S_{11}\right) \circ \left(y_2 - y\right) + \left(S_{22} - S_{12}\right) \circ \left(y - y_1\right)\right) \circ B_{11}$$

We must now be careful because we did not in fact calculate the gradient with respect to a single value within U_{11}, which is what we actually need. To do this you have to add together all the values within each grid block, then offset the different gradients with respect to each U matrix. Further simplification is difficult because both y and T-R vary from pixel to pixel, which limits combining matrices within a block as well as between the four neighboring blocks for each control point variable. Following the derivation pattern above we can obtain all four gradient functions for the U matrix.

$$\frac{dF}{dU_{ij}} = 2(T - R) \circ \left(\left(S_{21} - S_{11}\right) \circ \left(y_2 - y\right) + \left(S_{22} - S_{12}\right) \circ \left(y - y_1\right)\right) \circ B_{ij}$$

We now look at the derivatives with respect to the V matrix. It is the same as the above procedure until the derivative of the A matrices.

$$\frac{dA_{11}}{dV_{ij}} = -\left(x_2 - x\right)\frac{dy}{dV_{ij}}$$

$$\frac{dA_{12}}{dV_{ij}} = \left(x_2 - x\right)\frac{dy}{dV_{ij}}$$

$$\frac{dA_{21}}{dV_{ij}} = -\left(x - x_1\right)\frac{dy}{dV_{ij}}$$

$$\frac{dA_{22}}{dV_{ij}} = \left(x - x_1\right)\frac{dy}{dV_{ij}}$$

Then repeating the simplification process for R:

$$\frac{dR}{dV_{ij}} = \left(\left(S_{12} - S_{11}\right) \circ \left(x_2 - x\right) + \left(S_{22} - S_{21}\right) \circ \left(x - x_1\right)\right) \circ \frac{dy}{dV_{ij}}$$

Similarly, the D matrix expression simplifies based on the choice of V_{ij}.

$$\frac{dy}{dV_{ij}} = \frac{dD}{dV_{ij}} = B_{ij}[i, j]$$

This leaves a convenient and simple expression for all eight gradients:

$$Z_U = \left(S_{21} - S_{11}\right) \circ \left(y_2 - y\right) + \left(S_{22} - S_{12}\right) \circ \left(y - y_1\right)$$
$$Z_V = \left(S_{12} - S_{11}\right) \circ \left(x_2 - x\right) + \left(S_{22} - S_{21}\right) \circ \left(x - x_1\right)$$

$$W_U = K(T - R) \circ Z_U$$
$$W_V = K(T - R) \circ Z_V$$

$$\frac{dF}{dU_{ij}} = W_U \circ B_{ij}$$
$$\frac{dF}{dV_{ij}} = W_V \circ B_{ij}$$

We have generalized the constant term, formerly two, to allow for rescaling to account for changes caused by the imresize function.

Hessian

The hessian is the gradient of the gradient. Each grid block defines the motion of a set of pixels. Therefore, there are eight underlying variables which are interrelated, which match the eight gradients computed above. There are four general cases:

$$\frac{dF}{dU_{ij}} = W_U \circ B_{ij}$$

$$\frac{dF}{dV_{ij}} = W_V \circ B_{ij}$$

$$\frac{d^2F}{dU_{ij}\, dU_{\hat{i}\hat{j}}} = \frac{dW_U}{dU_{ij}} \circ B_{\hat{i}\hat{j}}$$

$$\frac{d^2F}{dV_{ij}\, dV_{\hat{i}\hat{j}}} = \frac{dW_V}{dV_{ij}} \circ B_{\hat{i}\hat{j}}$$

$$\frac{d^2F}{dV_{ij} dU_{\hat{i}\hat{j}}} = \frac{dW_U}{dV_{ij}} \circ B_{\hat{i}\hat{j}}$$

$$\frac{d^2F}{dU_{ij} dV_{\hat{i}\hat{j}}} = \frac{dW_V}{dU_{ij}} \circ B_{\hat{i}\hat{j}}$$

Dummy variables are used to distinguish between the first and second derivatives. For the matching derivatives (dUdU, dVdV), the Z terms are independent of the derivative leading to a simple hessian value which can be computed easily from the gradient.

$$W_U = K(T - R) \circ Z_U$$

$$W_V = K(T - R) \circ Z_V$$

$$\frac{dW_U}{dU_{ij}} = -K\frac{dR}{dU_{ij}} \circ Z_U$$

$$\frac{dW_v}{dV_{ij}} = -K\frac{dR}{dV_{ij}} \circ Z_V$$

For opposing derivatives (dUdV, dVdU), The cross-terms are not independent and the product rule must be used again. Substituting and simplifying:

$$\frac{dW_U}{dV_{ij}} = -K\frac{dR}{dV_{ij}} \circ Z_U + K(T - R) \circ \frac{dZ_U}{dV_{ij}}$$

$$\frac{dW_V}{dU_{ij}} = -K\frac{dR}{dU_{ij}} \circ Z_V + K(T - R) \circ \frac{dZ_V}{dU_{ij}}$$

$$Z_U = \left(S_{21} - S_{11}\right) \circ \left(y_2 - y\right) + \left(S_{22} - S_{12}\right) \circ \left(y - y_1\right)$$
$$Z_V = \left(S_{12} - S_{11}\right) \circ \left(x_2 - x\right) + \left(S_{22} - S_{21}\right) \circ \left(x - x_1\right)$$

$$\frac{dZ_U}{dV_{ij}} = \left(S_{21} - S_{11}\right) \circ \left(- \frac{dy}{dV_{ij}}\right) + \left(S_{22} - S_{12}\right) \circ \left(\frac{dy}{dV_{ij}}\right)$$
$$\frac{dZ_V}{dU_{ij}} = \left(S_{12} - S_{11}\right) \circ \left(- \frac{dx}{dU_{ij}}\right) + \left(S_{22} - S_{21}\right) \circ \left(\frac{dx}{dU_{ij}}\right)$$

$$\frac{dZ_U}{dV_{ij}} = \left(S_{22} - S_{12}\right) \circ B_{ij} - \left(S_{21} - S_{11}\right) \circ B_{ij}$$
$$\frac{dZ_V}{dU_{ij}} = \left(S_{22} - S_{21}\right) \circ B_{ij} - \left(S_{12} - S_{11}\right) \circ B_{ij}$$

$$\frac{dZ_U}{dV_{ij}} = \frac{dZ_V}{dU_{ij}} = \left(S_{22} - S_{12} - S_{21} + S_{11}\right) \circ B_{ij}$$

$$\frac{dW_U}{dV_{ij}} =- K \frac{dR}{dV_{ij}} \circ Z_U + K(T - R) \circ \left(S_{22} - S_{12} - S_{21} + S_{11}\right) \circ B_{ij}$$
$$\frac{dW_V}{dU_{ij}} =- K \frac{dR}{dU_{ij}} \circ Z_V + K(T - R) \circ \left(S_{22} - S_{12} - S_{21} + S_{11}\right) \circ B_{ij}$$

Regularization

We have two matrices, U and V, representing a series of control point positions. Further we define submatrices U11, U12 … V11, V12 … which represent the corners of each square. The components of each side are as follows:

$$x_1 = U_{12} - U_{11}$$
$$x_2 = U_{11} - U_{21}$$
$$x_3 = U_{22} - U_{12}$$
$$x_4 = U_{21} - U_{22}$$

$$y_1 = V_{12} - V_{11}$$
$$y_2 = V_{11} - V_{21}$$
$$y_3 = V_{22} - V_{12}$$
$$y_4 = V_{21} - V_{22}$$

We can now define the square of each side length using components.

$$S_1^2 = x_1^2 + y_1^2$$
$$S_2^2 = x_2^2 + y_2^2$$
$$S_3^2 = x_3^2 + y_3^2$$
$$S_4^2 = x_4^2 + y_4^2$$

Adding the four squares of the side lengths gives an area equal to the squares of the diagonals of the quadrilateral. This effectively measures how stretched out the square is, as this value is minimized when the quadrilateral is a square, given a constant area.

$$D = S_1^2 + S_2^2 + S_3^2 + S_4^2 - 4$$

Each side length is initially one, so the sum of the squares is equal to four. We set the cost function as a least-squares fit to the initial sum with coefficient K.

$$F(U, V) = KD^2$$

Starting with the gradient of the overall expression.

$$\frac{dF}{dU_{ij}} = 2KD\frac{dD}{dU_{ij}}$$
$$\frac{dF}{dV_{ij}} = 2KD\frac{dD}{dV_{ij}}$$

Each Uij or Vij has their own gradient based on the signs in the expression.

$$\frac{dD}{dU_{11}} = 2x_2 - 2x_1$$
$$\frac{dD}{dU_{12}} = 2x_1 - 2x_3$$
$$\frac{dD}{dU_{21}} = 2x_4 - 2x_2$$
$$\frac{dD}{dU_{22}} = 2x_3 - 2x_4$$

$$\frac{dD}{dV_{11}} = 2y_2 - 2y_1$$
$$\frac{dD}{dV_{12}} = 2y_1 - 2y_3$$
$$\frac{dD}{dV_{21}} = 2y_4 - 2y_2$$
$$\frac{dD}{dV_{22}} = 2y_3 - 2y_4$$

The hessian terms are then:

$$\frac{d^2F}{dU_{ij}\,dU_{\widehat{ij}}} = 2KD\left(\frac{dD}{dU_{ij}}\frac{dD}{dU_{\widehat{ij}}} + \frac{d^2D}{dU_{ij}\,dU_{\widehat{ij}}}\right)$$
$$\frac{d^2F}{dV_{ij}\,dV_{\widehat{ij}}} = 2KD\left(\frac{dD}{dV_{ij}}\frac{dD}{dV_{\widehat{ij}}} + \frac{d^2D}{dV_{ij}\,dV_{\widehat{ij}}}\right)$$

$$\frac{d^2F}{dV_{ij}dU_{\widehat{ij}}} = 2KD\frac{dD}{dV_{ij}}\frac{dD}{dU_{\widehat{ij}}}$$
$$\frac{d^2F}{dU_{ij}dV_{\widehat{ij}}} = 2KD\frac{dD}{dU_{ij}}\frac{dD}{dV_{\widehat{ij}}}$$

The second derivative term is equal to zero if the indices are on opposite corners, four if the indices are the same, and negative two if the indices are on adjacent corners.

Appendix B: MATLAB Code for Craniotomy Phantom

```
function [video, field, mask] = generate_phantom_video()
%GENERATE_PHANTOM_VIDEO generates a video of a pulsating phantom
%
%   Usage:
%   [video, field] = generate_phantom_video()
%
%   Outputs:
%   video       phantom video
%   field       deformation field
%   mask        craniotomy mask
%

% Video parameters
period = 30;
amplitude = 8;

% Create checkerboard image
[image, mask] = generate_phantom;

% Generate a distance grid the frame size
xvals = 1:768;
yvals = 1:1024;
[X, Y] = meshgrid(yvals, xvals);
X = X - mean(X, 'all');
Y = Y - mean(Y, 'all');

% Generate a gaussian amplitude window
fs = 2 * pi / (768 - 1);
k = fs.^2 / 2;
F = exp(-k * (X.^2 + Y.^2));

% Generate the deformation amplitude
[DX, DY] = gradient(F);
DX = DX ./ max(DX, [], 'all');
DY = DY ./ max(DY, [], 'all');
D = cat(3, DX, DY);

% Generate the scaling time series
idxs = 0:150;
cphases = pi * idxs / period;
rphases = 0.2 * pi * idxs / period;
scalings = sin(rphases) + 0.5 * abs(sin(cphases));
```

```
scalings = amplitude * rescale(smooth(scalings, 5));

% For the number of specified frames
for fidx = 1:151

    % Calculate the deformation
    DF = scalings(fidx) * D .* mask;
    video(:,:,fidx) = imwarp(image, DF);
    field(:,:,fidx,:) = DF;

end

end

function [phantom, craniotomy] = generate_phantom()
%GENERATE_PHANTOM Creates a synthetic thermal base image
%
%   Usage:
%   [image, craniotomy] = generate_phantom()
%
%   Outputs:
%   iamage          reference image
%   craniotomy      soft mask which contains the craniotomy
%

% Create craniotomy
craniotomy = zeros(768, 1024);
craniotomy(384:385, 512:513) = 1;
radius = 370;
H = fspecial('disk',radius);
craniotomy = imfilter(craniotomy, H, 'same') > 0;
craniotomy = imgaussfilt(double(craniotomy), 1);

% Create the foreground
foreground = checkerboard(64, 6, 8) > 0;
foreground = checkerboard(32, 12, 16) > 0;
foreground = 0.5 * foreground + 0.5;
% foreground = imgaussfilt(foreground, 1);
foreground = imgaussfilt(foreground, 2, 'FilterSize', 17);
foreground = craniotomy .* foreground;

% Create the background
xvals = 1:768;
yvals = 1:1024;
```

```
[X,Y] = meshgrid(yvals,xvals);
background = 0.5 * sin((X + Y) / 2);

% Merge the background and foreground
phantom = craniotomy .* foreground + ~craniotomy .* background;

end
```

Appendix C: Hyperparameter Optimization

A grid search parameterization approach was employed for hyperparameter optimization. All methods had two hyperparameters that needed optimization except for phase correlation, where the standard deviation of the gaussian high pass filter was the only parameter to optimize. For bispline registration, the regularization parameter and the number of optimization steps were chosen. For demons registration, the smoothness parameter and number of pyramid levels were chosen. For the optical flow methods, the smoothness coefficient and noise threshold were chosen for the Horn-Schunck and Lucas-Kanade methods, respectively. As both optical flow methods were repeated a number of times on the data, the number of repeats was also optimized. For the band-pass filter, the parameters have already been specified in the literature so these were not changed.

Grid search was performed by running all methods for each parameter combination in the search space. Data from all patients were used, but were temporally downsampled to 0.1 Hz to make computation times reasonable. Phase correlation was optimized first, and these parameters were used for optimization of the remaining nonrigid methods except for bispline registration, which does not require phase correlation. The IMMSE, PSNR, and SSIM were calculated for all patients, parameter combinations, and methods. Results from each patient were then concatenated for each parameter combination, and compared using a nonparametric statistical approach specified below.

A one-sided Wilcoxon signed-rank test was performed on each parameter combination for each metric (SSIM, PSNR, IMMSE) to test if it produces better results (higher SSIM, PSNR, or lower IMMSE) across all patients than all other parameter combinations for that method. The negative logarithm of the median probability value of the parameter combination vs. all other combinations was calculated for each parameter combination, and interpreted as an overall performance score. The parameter combination with the highest harmonic mean of the IMMSE, SSIM, and PSNR scores was chosen for each method. If the best parameter combination was on the edge of the search grid, the parameter space was modified so that the optimal combination is centered, and the analysis was repeated until a non-border optimal parameter combination was found.

This approach was taken over the more standard approach of averaging performance of methods across all subjects in order to account for large intra-subject variance observed in this dataset. Furthermore, as each metric is a relative measure of image quality, it is not generally recommended to directly compare the metrics across subjects. Our matched, non-parametric techniques accomplish this while being less sensitive to outliers. The final parameters and graphs for the search results are shown for each method below:

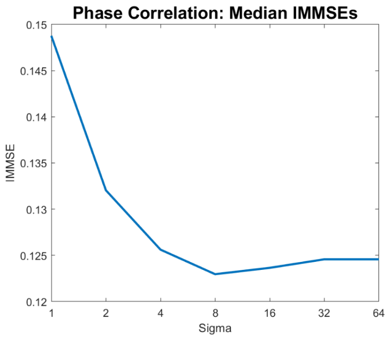
Phase Correlation: Median IMMSEs
IMMSE
0.15
0.145
0.14
0.135
0.13
0.125
0.12
1 2 4 8 16 32 64
Sigma

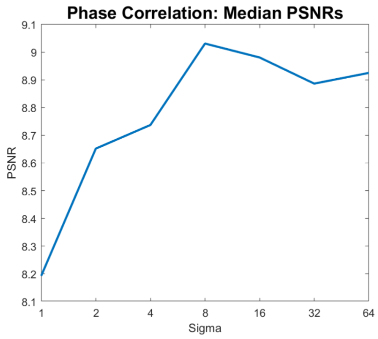
Phase Correlation: Median PSNRs
PSNR
9.1
9
8.9
8.8
8.7
8.6
8.5
8.4
8.3
8.2
8.1
1 2 4 8 16 32 64
Sigma

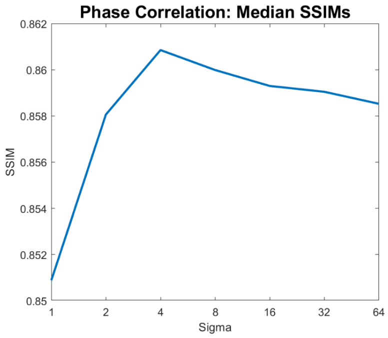
Phase Correlation: Median SSIMs
SSIM
0.862
0.86
0.858
0.856
0.854
0.852
0.85
1 2 4 8 16 32 64
Sigma

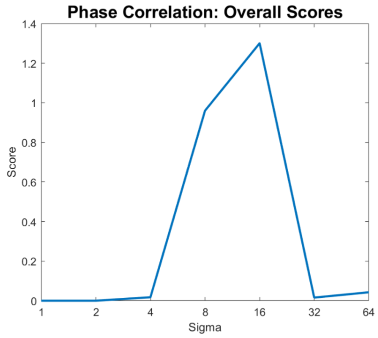
Phase Correlation: Overall Scores
Score
1.4
1.2
1
0.8
0.6
0.4
0.2
0
1 2 4 8 16 32 64
Sigma

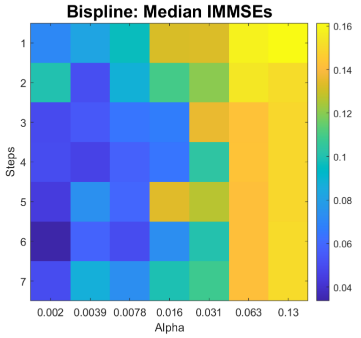
Bispline: Median IMMSEs
Steps
1 2 3 4 5 6 7
0.002 0.0039 0.0078 0.016 0.031 0.063 0.13
Alpha
0.16
0.14
0.12
0.1
0.08
0.06
0.04

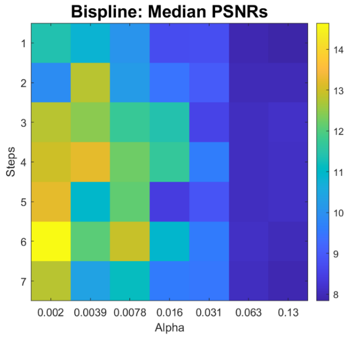
Bispline: Median PSNRs
Steps
1 2 3 4 5 6 7
0.002 0.0039 0.0078 0.016 0.031 0.063 0.13
Alpha
14
13
12
11
10
9
8

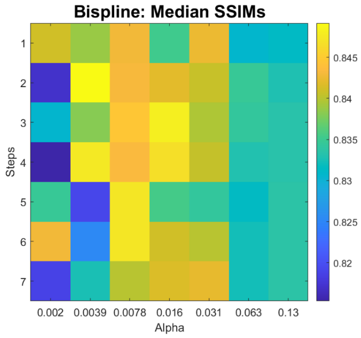
Bispline: Median SSIMs
Steps
1 2 3 4 5 6 7
0.002 0.0039 0.0078 0.016 0.031 0.063 0.13
Alpha
0.845
0.84
0.835
0.83
0.825
0.82

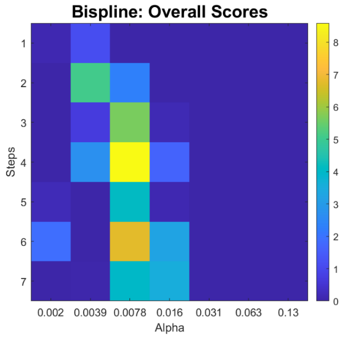
Bispline: Overall Scores
Steps
1 2 3 4 5 6 7
0.002 0.0039 0.0078 0.016 0.031 0.063 0.13
Alpha
8
7
6
5
4
3
2
1
0

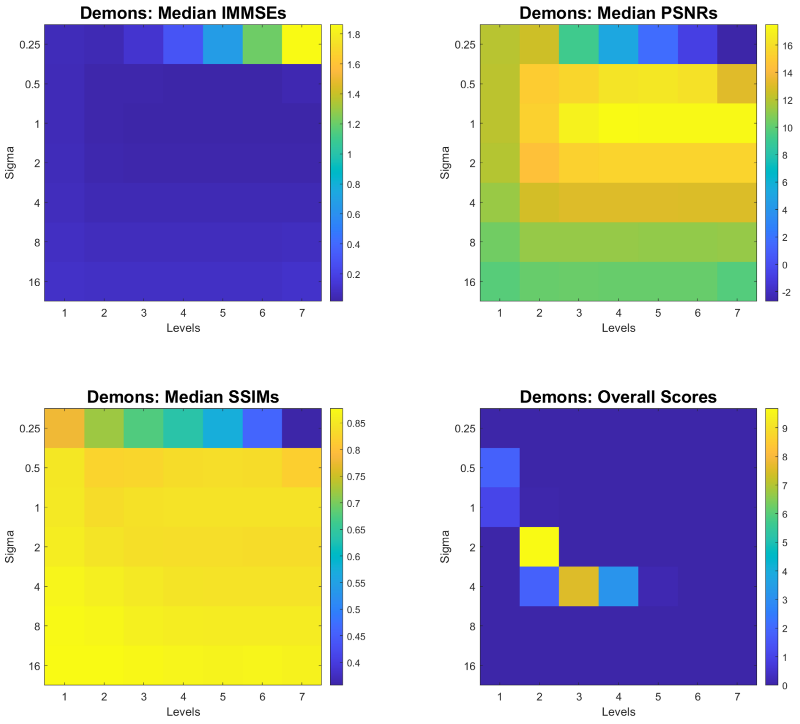
Demons: Median IMMSEs
Demons: Median PSNRs
Demons: Median SSIMs
Demons: Overall Scores
Sigma
Levels
0.25
0.5
1
2
4
8
16
1 2 3 4 5 6 7

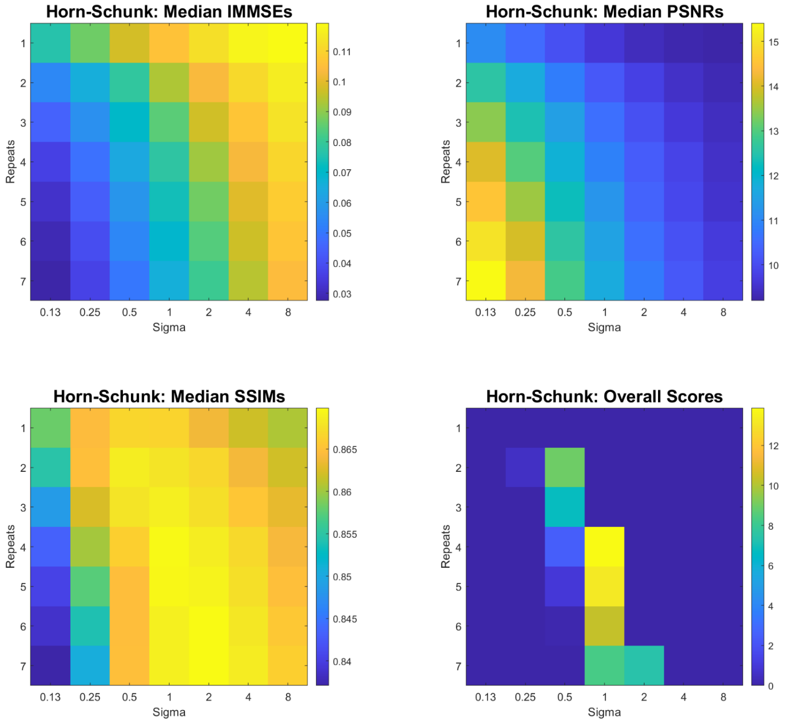
Horn-Schunk: Median IMMSEs
Horn-Schunk: Median PSNRs
Horn-Schunk: Median SSIMs
Horn-Schunk: Overall Scores
Repeats
Sigma
1 2 3 4 5 6 7
0.13 0.25 0.5 1 2 4 8

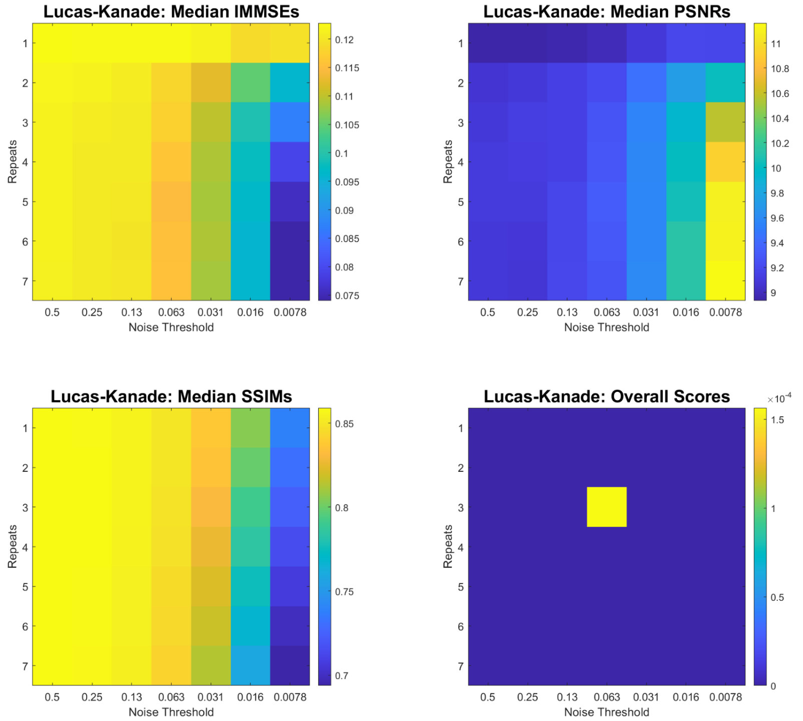

Figure S1. Hyperparameter Search Results. The results of the grid hyperparameter search are shown in four plots for each method. The median IMMSE (top left), PSNR (top right), and SSIM (bottom right) across all frames and patients is illustrated as a function of the parameter search space. Finally, the overall score, computed as specified above, is shown (bottom right).

Optimal parameters were identified for each method, however some methods were more difficult to parameterize than others. For the phase correlation, bispline, and demons methods, the IMMSE was typically anticorrelated with the SSIM and the PSNR, as expected. However this pattern was not observed for the optical flow techniques. For the Horn-Schunk method, the SSIM was correlated with the IMMSE and anti-correlated with the PSNR, while in the Lucas-Kanade comparison the SSIM was not correlated with either other metric. This indicates that there are no parameter combinations within the search space which simultaneously value matching individual pixel intensities while preserving local image contrast, and therefore the selection of optical hyperparameters for these methods is challenging.

Final parameters for each method are listed here. Phase correlation: sigma was chosen as 16 from an exponential range of one to sixty-four. Bispline: the number of optimization steps was chosen as 4 from a linear range of one to seven, and the regularization parameter alpha was chosen as one divided by 128 from an exponential range of one eight to one divided by 512. Demons registration: the smoothing parameter sigma was chosen as two from an exponential range of one fourth to 16, and the number of pyramid levels was chosen as two from a linear range of one to seven. Horn-Schunk: the smoothing parameter was chosen as one from an exponential range of one eight to eight, and the number of times to repeat the algorithm on the video was chosen as four from a linear range of one to seven. Lucas-Kanade: the noise threshold was chosen as one sixteenth from an exponential range of one half to one divided by 128, and the number of times to repeat the algorithm on the video was chosen as three from a linear range of one to seven.
